# A new *Synersaga* species from Cambodia (Lepidoptera, Lecithoceridae), with a world catalogue of the genus

**DOI:** 10.3897/zookeys.187.2660

**Published:** 2012-04-27

**Authors:** Kyu-Tek Park, Yang-Seop Bae

**Affiliations:** 1 The Korean Academy of Science and Technology, Seungnam, Gyunggi, 463-808 Korea; McGuire Center for Lepidoptera and Biodiversity, University of Florida, Gainesville, FL 32611 USA; 2 Division of Life Sciences, University of Incheon, Incheon, 406-772 Korea

**Keywords:** Lepidoptera, Lecithoceridae, *Synersaga*, new species, Cambodia, taxonomy

## Abstract

A new species of the genus *Synersaga* Gozmány, *Synersaga mondulkiriensis* **sp. n.**, is described from Cambodia. The genus is diagnosed, and a global catalogue for the genus is provided.

## Introduction

The family Lecithoceridae (Lepidoptera, Gelechioidea) is characterized by the very long antenna, usually longer than the forewing, and the male genitalia with gnathos bent downwards or absent. These characters are useful to differentiate from other gelechioid-moths. With respect to Lecithoceridae biology, larvae are known to feed on dead plant materials. A few Australian species have been reported to be reared on leaf litters of eucalypt (Common 1996). Recently, Komai et al. (2011) reported that two species of Lecithocerinae (*Homaloxestis myeloxesta* Meyrick, 1932 and *Lecithocera thiodora* (Meyrick, 1914)) and three species of Torodorinae (*Athymoris martialis* Meyrick, 1935, *Deltoplastis apostatis* (Meyrick, 1932), and *Halolaguna sublaxata* Gozmány, 1978) were reared from dead leaves of several unknown broadleaved trees in Japan. The family is mostly distributed in the Oriental and Australian Regions, around 1,200 described species (van Nieukerken et al. 2011).

*Synersaga* Gozmány, 1978 is a small genus belonging to the subfamily Lecithocerinae that comprises six species only in the Oriental Region: the type species, *Synersaga pseudocathara* (Diakonoff, 1952) described from Myanmar, and five more species from East and Southeast Asia (Gozmány 1978; Park 2000, 2009; Park et al. 2007). Herein a new species, *Synersaga mondulkiriensis* sp. nov., is described from Cambodia. Moths have usually unicolorous forewing with yellowish-brown to dark-fuscous ground color.

The genus is allied to *Lecithocera* Herrich-Schäffer, 1853 and is defined by the combination of following characters: vein R_3_ on the forewing is separate or connate and the male genitalia have the cucullus fairly elongated and usually expanded distally, and well- developed caudal processes of the juxta. On the other hand, for several species of *Lecithocera* known from Sri Lanka, which have male genitalia resembling *Synersaga*, e.g. *Lecithocera capnaula* Meyrick, 1911, *Lecithocera haemylopsis* (Meyrick, 1911), *Lecithocera nubigena* (Meyrick, 1911), *Lecithocera paroena* (Meyrick, 1906), and *Lecithocera paroristis* (Meyrick, 1911), the generic placement should be reconsidered by examining the forewing venation.

## Material and methods

The present study is based on recent material collected by the authors in Cambodia, from the result of an entomological expedition to Cambodia by the Environmental Ministry, Korea. The wingspan is measured from the left wing apex to the right wing apex, including fringe. The color standard for the description of adults follows Kornerup and Wanscher (1978). Types are deposited in the University of Incheon, Korea (UIK) on indefinite loan from Cambodia. Abbreviations for museums:HMNH= Hungarian Museum of natural History, Budapest, Hungary; KNA= Korea National Arboretum, Pocheon, Korea; UIK= University of Incheon, Korea; OPU= Osaka Prefectural University, Osaka, Japan; NRS= Naturhistoriska Riksmuseet, Stockholm, Denmark.

## Taxonomic Accounts

### Genus Synersaga

Gozmány, 1978

http://species-id.net/wiki/Synersaga

Synersaga Gozmány 1978: 141; Wu 1997: 174; Park et al. 2007: 206; Park 2009: 2.
Lecithocera pseudocathra Diakonoff 1951: 76 - Type species. Type locality: Myanmar
= Anamimnesis Gozmány 1978: 143. Type species: *Anamimnesis bleszynskii*Gozmany 1978: 143 (synonimized by Park 2000).

#### Note.

*Synersaga* is characterized by the forewing characters: forewing slightly broader distally with round apex, evenly colored, with yellowish brown or blackish ground color; venation with R_3_ free or connate with R_4+5_; M_3_ and CuA_2_ short-stalked or connate. However, the forewing color patterns of the known species are very similar to each other and they can be differentiated from one another by the shape of the uncus and the caudal processes of the juxta in the male genitalia. The abdominal tergites are densely spinose, and the seventh tergite is uniquely specialized, produced laterally with a sclerotized anterior margin.

#### World catalogue of *Synersaga*

*Synersaga bleszynskii* (Gozmány, 1978: 143) China

TL (Type locality): Chekiang, China. Type in HMNH.

Fig.: Gozmány (1978, Taf. 8, 37, Fig. 86; Park (2000, Figs 20, 20a)

*Synersaga caradjai* (Gozmány, 1978: 143) Taiwan

TL: Kosempo, Taiwan. Type in HNHM.

Fig.: Gozmány (1978, Taf. 8, 37, Fig. 85)

*Synersaga kuni* Park, 2007: 206 Vietnam

TL: Cuc Phoung, N. Vietnam. Type in KNA.

Fig.: Park et al. (2007, Figs 8, 17, 17a)

*Synersaga mondulkiriensis* sp. n. Cambodia

TL: Mondulkiri, Cambodia. Type in UIK.

Fig.: Park & Bae (2012, Figs 4–12 )

*Synersaga nigriptera* Park, 2007: 208 Vietnam

TL: Babe, N. Vietnam. Type in KNA.

Fig.: Park et al. (2007, Figs 9, 18, 18a, 22)

*Synersaga phuruaensis* Park, 2009:2 Thailand

TL: Loei, China. Type in OPU.

Fig.: Park (2009, Figs 4-6, 8, 8a, 10)

*Synersaga pseudocathra* (Diakonoff, 1951: 76)* Myanmar

Ark. Zool. 1951, 3: 76. TL: Kambaiti, Myanmar. Type in NRS.

Fig.: Diakonoff (1951, Figs 13 (male), 15 &16 (female))

### Synersaga mondulkiriensis sp. n.

urn:lsid:zoobank.org:act:839DA14C-9E99-4CEC-A7A0-24E0826F8454

http://species-id.net/wiki/Synersaga_mondulkiriensis

Figs 1 12

#### Type material.

Holotype ♂ – Cambodia, Prov. Mondulkiri, Seima Biodiversity Conservation Area, 12°57'N, 107°10'E, 3–8 July 2009 (Bae & Chae), gen. slide no. CIS-6072/Park. Paratype – 3♂, 1♀, same locality, 7 Oct. 2010.

#### Diagnosis.

This new species is similar to *Synersaga pseudocathara* from Myanmar, and *Synersaga kuni* and *Synersaga nigriptera* from Vietnam in the external and male genitalic characters. It can be distinguished from them by the shape of cucullus and the caudal processes of the juxta in the male genitalia. The caudal processes of the juxta of the new species are similar to those of *Synersaga nigriptera*, but longer and arched inwardly, and the distal portion of the cucullus is more or less clavate.

#### Description.

**Figures 1–9.**
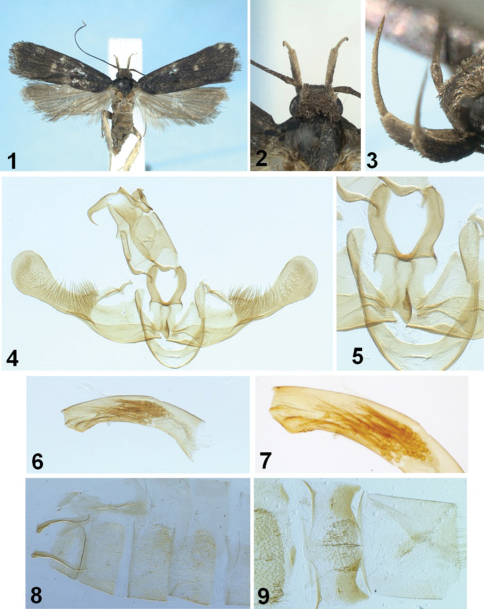
*Synersaga mondulkiriensis* sp. n., holotype **1** adult, holotype **2** head in dorsal view **3** labial palpus **4** male genitalia **5** close-up of juxta **6** aedeagus **7** close-up of cornuti **8** 1^st^-4^th^ abdominal segments **9** close-up of 6^th^–8^th^ abdominal segments. Scale bar: 1 mm.

Adult (Figures 1, 2, 3). Wingspan, 17–18 mm. Head and thorax dark fuscous dorsally. Antenna dark fuscous throughout, relatively thick. Second segment of labial palpus fairly thickened, dark fuscous on outer surface with orange white apex, orange white on inner surface; 3^rd^ segment slender, as long as 2^nd^ segment, orange white all around. Forewing covered with dark fuscous scales throughout; two blackish discal spots well developed: one in middle, the other larger one at end of cell; apex rounded; termen slightly concave medially; venation with R_1_ arising from middle of cell; R_2_ nearer to R_3_ than R_1_ at base; R_3_ free; R_4_ and R_5_ stalked for basal 3/5 length; R_5_ reaching just beyond apex; M_3_ arising from half between M_2_ and CuA_1+2_ at base; CuA_1_ and CuA_2_ stalked for basal 1/5. Hindwing broader than forewing, pale brownish orange; apex more or less obtuse; termen sinuate; fringe concolorous, with narrow orange white basal line; venation with M_3_ and CuA_1_ short stalked. Hind tibia clothed with orange gray scales.

**Male genitalia** (Figures 4, 5, 6, 7). Uncus broad, short, obtuse, not exceeding basal stalk of gnathos, with small median lobe on caudal margin. Median process of gnathos strongly bent beyond middle, with acute apex. Valva broad basally, with triangular process near base on costa; costa gently concave; ventral margin gently arched outward in basal half; cucullus elongate, broadly expanded with round outer margin; dense long setae in basal half of cucullus, fairly setose beyond. Juxta with caudal processes long, gently arched inward, while the processes in *Synersaga nigriptera* nearly straight, clavate. Aedeagus gently curved, shorter than valva+cucullus, with finely dentate along ventral and dorsal margins apically; cornuti consist of a series of numerous needle-like cornuti. Abdominal segments in Figures 8 and 9.

**Figures 10–12.**
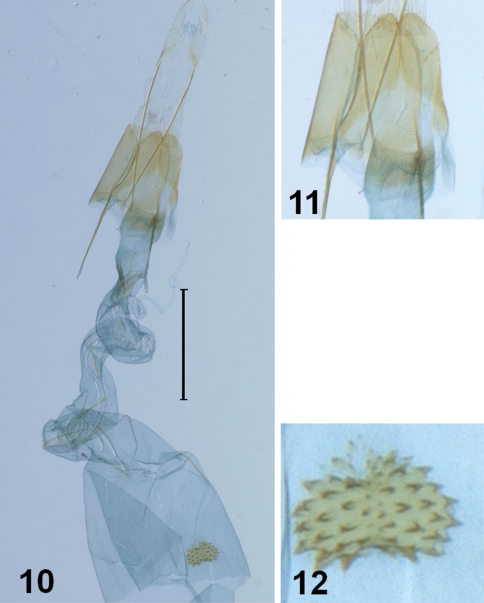
Female genitalia of *Synersaga mondulkiriensis* sp. n. **10** genitalia **11** 8^th^ segment **12** genitalia. Scale bar: 1 mm.

**Female genitalia** (Figures 10, 11, 12). Similar to those of *Synersaga nigriptera*. Caudal margin of eighth abdominal sternite with deep Y-shaped medial emargination. Dorsal surface of ostial plate with dense spinules; caudal margin of ostium bursae concave. Antrum weakly sclerotized, cup-shaped. Ductus bursae coiled twice, slightly longer than corpus bursae, nearly same width throughout, with several needle-like spines internally. Corpus bursae elongate; signum a semiovate plate denticulate throughout.

#### Distribution.

Cambodia (Mondulkiri).

#### Etymology.

The species name is derived from the type locality.

## Supplementary Material

XML Treatment for Synersaga

XML Treatment for Synersaga mondulkiriensis

## Footnotes

Figure by Diakonoff (1951) was erroneously numbered: Fig. 14 is that of *Lecithocera pellax* Meyrick; and Gozmány (1978) cited erroneously “Daikonoff (1951)” as “Diakonoff (1952)”.

## References

[B1] Diakonoff A (1951) Entomological results from the Swedish expedition 1934 to Burma and British India. Lepid. Microlepidoptera 1. Arkiv for Zoologi. (2)3(6): 59–94.

[B2] Gozmány L (1978)Lecithoceridae.In: Amsel HG, Gregor F, Reisser H (Eds) Microlepidoptera Palaeartica. Vol. 5. Georg Fromme & Co., Wien, 306 pp.

[B3] Komai F Yoshiyasu Y Nasu Y Saito T (2011) A guide to the Lepidoptera of Japan. Tokai University Press, Kanagawa, 1305 pp.

[B4] Kornerup A Wanscher JH (1978) Methuen Handbook of Colour. 2nd ed. Methuen & Co., London, 252 pp.

[B5] Nieukerken EJ van Kaila L Kitching IJ Kristensen NP Lees DC Minet J Mitter C Mutanen M Regier JC Simonsen TJ Wahlberg N Yen S-H Zahiri R Adamski D Baixeras J Bartsch D Bengtsson BÅ Brown JW Bucheli SR Davis DR De Prins J De Prins W Epstein ME Gentili-Poole P Gielis C Hättenschwiler P Hausmann A Holloway JD Kallies A Karsholt O Kawahara AY Koster SJC Kozlov M Lafontaine JD Lamas G Landry J-F Lee S Nuss M Park K-T Penz C Rota J Schintlmeister A Schmidt BC Sohn J-C Solis MA Tarmann GM Warren AD Weller S Yakovlev RV Zolotuhin VV Zwick A (2011) Order Lepidoptera Linnaeus, 1758. In: Zhang Z -Q. (Ed.), Animal biodiversity: An outline of higher-level classification and survey of taxonomic richness.Zootaxa 3148: 212-221 http://www.mapress.com/zootaxa/2011/f/zt03148p221.pdf

[B6] Park KT (2009) Two new species of the genus *Tisis* Walker and *Synersaga* Gozmány (Lepidoptera, Lecithoceridae) from Thailand. Tropical Lepidoptera Research 19: 1-3

[B7] Park KT (2000) Lecithoceridae (Lepidoptera) of Taiwan (II): Sufamily Lecithocerinae: Genus *Lecithocera* Herrich-Schäffer and its allies. Zoological Studies 39: 360-374

[B8] Park KT Kim MY Kim Sora Cha MY Byun BK Nguyen C (2007) Lecithoceridae of Vietnam I. Genera *Homaloxestis* Meyrick and *Synersaga* Gozmány. Journal of Asia Pacific Entomology 10: 201-209 doi: 10.1016/S1226-8615(08)60354-4

[B9] Wu C (1997) Lepidoptera Lecithoceridae. Fauna Sinica, Insecta, 7. Science Press, Beijing, 302 pp.

